# Incidental giant thymoma—a reminder of the importance of a global look of the imaging scans

**DOI:** 10.1093/jscr/rjad084

**Published:** 2023-03-04

**Authors:** Filipa Policarpo, Mariana Antunes, Magda Alvoeiro, Francisco Alvoeiro

**Affiliations:** General Surgery, Centro Hospitalar Lisboa Ocidental, Lisboa, Portugal; Thoracic Surgery, Centro Hospitalar Lisboa Norte, Lisboa, Portugal; Thoracic Surgery, Centro Hospitalar Lisboa Norte, Lisboa, Portugal; Thoracic Surgery, Centro Hospitalar Lisboa Norte, Lisboa, Portugal

**Keywords:** radiology, surgical oncology, thoracic surgery, thymoma

## Abstract

A 49-year-old female patient, without previous medical history, underwent a thoracic CT due to SARS-CoV2 infection. This exam revealed a heterogeneous mass in the anterior mediastinum with 11 × 8.8 cm in close contact with main thoracic vessels and pericardium. Surgical biopsy documented a B2 thymoma. This clinical case reminds the importance of a systematic and global look of the imaging scans. Years before the thymoma diagnosis, the patient underwent a shoulder X-ray due to musculoskeletal pain, where an irregular shape of the aortic arch was visible, probably related to the growing mediastinal mass. An earlier diagnosis would allow a complete mass resection without such extensive surgery and less morbidity.

## INTRODUCTION

Thymomas are the most common primary tumor of the mediastinum in adults [1, 2].

Despite its usual slow-growing pattern, thymomas may require an extensive dissection and aggressive treatment in order to achieve its complete resection—the most important prognostic factor for local control and survival.

This clinical case reminds the importance of a systematic and global look of the imaging scans and its impact on early diagnosis and treatment.

## CASE REPORT

A former smoker (20 pack-years) 49-year-old female patient, without relevant previous medical history, underwent a thoracic CT due to a SARS-CoV2 infection (video 1). This exam revealed a heterogeneous mass in the anterior mediastinum with 11 × 8.8 cm, with calcifications and necrotic areas, in close contact with the aortic arch, supra-aortic branches, main pulmonary artery, left venous brachiocephalic trunk and pericardium. CT scan also showed a 46 × 20-mm pleural mass. Surgical biopsy of the mass documented a B2 thymoma.

After recovery from SARS-CoV2 infection, the patient remained asymptomatic and the only sign on the physical exam was a slight jugular venous engorgement.

The patient was referred to a thoracic oncology center and proposed to induction chemotherapy due to tumor size and vessel proximity. Given the significant mass reduction, surgery was proposed. The patient was submitted to en bloc thymectomy, pleuropneumonectomy, partial resection of the left venous brachiocephalic trunk, partial resection of diaphragm and pericardium, and pericardial reconstruction. Postoperative period went without major complications, and the patient was discharged 22 days after surgery.

Pathologic analysis of the mass confirmed the diagnosis of a B2 thymoma stage IVa, which is completely resected. In the postoperative multidisciplinary meeting, it was decided to propose adjuvant radiotherapy.

## DISCUSSION

Thymomas are the most common primary tumor of the mediastinum in adults [1, 2]. Its characteristic indolent growth pattern helps to explain the lack of symptoms and the paucity of findings on the physical examination observed in this clinical case.

In this particular case, preoperative chemotherapy was important to reduce the compressive effects over the vascular structures and allows a safer procedure. However, due to the large size and proximity to major blood vessels, an extensive dissection was needed to ensure complete mass resection, given its impact as a prognostic factor.

**Figure 1:**
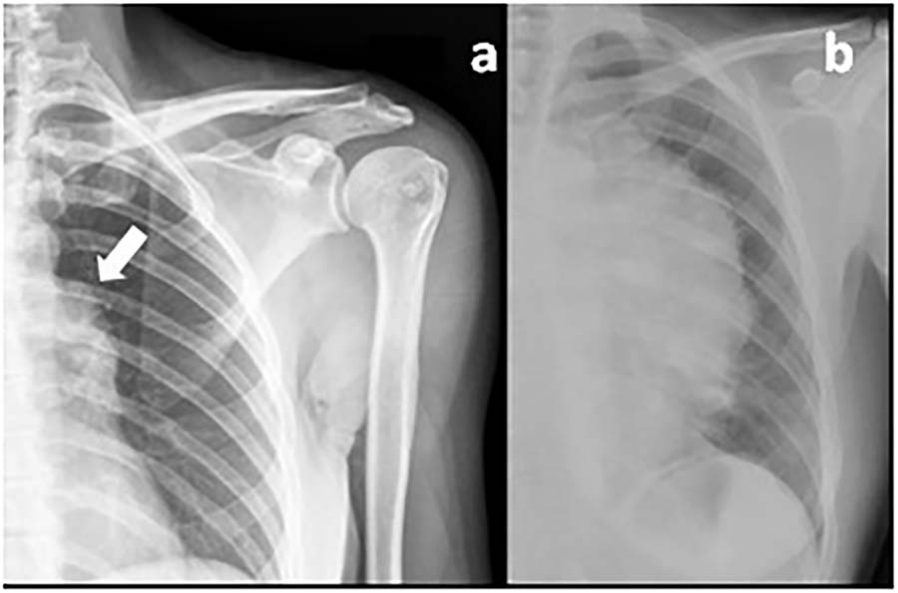
Previous shoulder X-ray **(a)** showing an irregular shape of the aortic arch (arrow) probably related to the mediastinal mass in comparison with a recent chest X-ray **(b)** showing the giant thymoma.

The curious aspect of this clinical case reminds us of the importance of a systematic and global look of the imaging scans. Years before the thymoma diagnosis, the patient underwent a shoulder X-ray (Fig. 1) due to localized musculoskeletal pain. In this image, an irregular shape of the aortic arch was visible, probably related to the growing mediastinal mass. An earlier diagnosis would perhaps allow a complete mass resection without such extensive surgery and less morbidity for the patient.

## Supplementary Material

Video_1_rjad084

## Data Availability

All the data are presented in the article and supplementary material.
